# Targeted reduction of the EGFR protein, but not inhibition of its kinase activity, induces mitophagy and death of cancer cells through activation of mTORC2 and Akt

**DOI:** 10.1038/s41389-017-0021-7

**Published:** 2018-01-23

**Authors:** Rajasekhara Reddy Katreddy, Lakshmi Reddy Bollu, Fei Su, Na Xian, Shivangi Srivastava, Rintu Thomas, Yubing Dai, Bing Wu, Yunlu Xu, Michael A. Rea, James M. Briggs, Qingyuan Zhang, Xiongbin Lu, Gangxiong Huang, Zhang Weihua

**Affiliations:** 1 Department of Biology and Biochemistry, College of Natural Sciences and Mathematics, University of Houston, Houston, TX 77204 USA; 2 Immunotherapy Institute, Fujian Medical University, University Town, Fuzhou, Fujian 350122 China; 3 Department of Medical Oncology, Cancer Hospital of Harbin Medical University, Harbin Medical University, 150081 Harbin, Heilongjiang China; 4 Department of Cancer Biology, University of Texas MD Anderson Cancer Center, Houston, TX 77030 USA; 5 Suzhou Institute, Xi’an Jiaotong University, Suzhou, Jiangsu 215123 China

## Abstract

The oncogenic epidermal growth factor receptor (EGFR) is commonly overexpressed in solid cancers. The tyrosine kinase activity of EGFR has been a major therapeutic target for cancer; however, the efficacy of EGFR tyrosine kinase inhibitors to treat cancers has been challenged by innate and acquired resistance at the clinic. Accumulating evidence suggests that EGFR possesses kinase-independent pro-survival functions, and that cancer cells are more vulnerable to reduction of EGFR protein than to inhibition of its kinase activity. The molecular mechanism underlying loss-of-EGFR-induced cell death remains largely unknown. In this study, we show that, unlike inhibiting EGFR kinase activity that is known to induce pro-survival non-selective autophagy, downregulating EGFR protein, either by siRNA, or by a synthetic EGFR-downregulating peptide (Herdegradin), kills prostate and ovarian cancer cells via selective mitophagy by activating the mTORC2/Akt axis. Furthermore, Herdegradin induced mitophagy and inhibited the growth of orthotopic ovarian cancers in mice. This study identifies anti-mitophagy as a kinase-independent function of EGFR, reveals a novel function of mTORC2/Akt axis in promoting mitophagy in cancer cells, and offers a novel approach for pharmacological downregulation of EGFR protein as a potential treatment for EGFR-positive cancers.

## Introduction

The epidermal growth factor receptor (EGFR) is oncogenic receptor tyrosine kinase that is often overexpressed/overactivated in cancers of epithelial origin, and drugs targeting the tyrosine kinase activity of EGFR have been developed as putative therapeutics to treat such malignancies. Although many types of cancer appear to depend upon upregulation of EGFR function for disease progression, EGFR tyrosine kinase inhibitors (TKI) have shown only transient clinical efficacy^1–4^. Furthermore, many EGFR-positive cancers, such as prostate cancer and ovarian cancer, are innately resistant to TKI^5,6^.

Studies over the past few years have revealed that EGFR promotes cancer cell survival through mechanisms that are independent of its tyrosine kinase activity^7–9^. Thus, an understanding of the mechanism(s) underlying EGFR’s kinase-independent (KID) functions offers great potential for the development of effective therapeutic approaches for cancer treatment. This possibility is strongly supported by the divergent responses of cancer cells to EGFR TKIs, vs. downregulation of EGFR protein. EGFR TKIs often cause growth arrest associated with non-selective, pro-survival autophagy^10–12^; however, loss-of-EGFR protein leads to severe autophagic cell death that could be rescued by a kinase-dead EGFR^7^, which suggests that the tyrosine kinase-dependent (KD) function of EGFR predominantly regulates cell proliferation, whereas the KID function of EGFR has a major role in promoting cancer cell survival. One important outstanding question regarding KD and KID functions of EGFR is that why TKI induced autophagy is pro-survival whereas loss-of-EGFR-induced autophagy is lethal. Answers to this question may reveal the core mechanism(s) underlying the KID pro-survival function of EGFR and should reveal new targets for the treatment of EGFR-dependent cancers.

In this study, using prostate and ovarian cancer cells, by comparing the autophagic phenotypes induced by EGFR TKI and by reduction of EGFR protein, we found a unique kinase-independent pro-survival function of EGFR, which is repression of selective mitophagy by inhibiting the mTROC2/Akt axis.

## Results

### Loss-of-EGFR, but not inhibition of its kinase activity, induced selective mitophagy

**Fig. 1:**
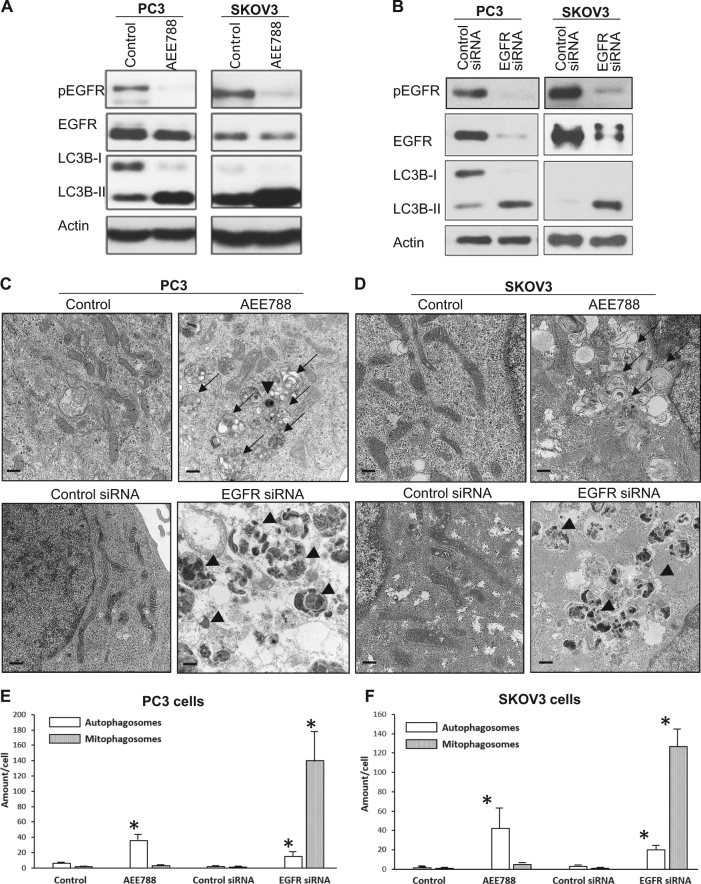
Loss-of-EGFR, but not inhibition of its kinase activity, induced mitophagy. **a** Western blot revealed that AEE788 significantly inhibited the phosphorylation of EGFR and elevated the level of an autophagy marker of LC3B-II. **b** Western blot revealed that EGFR knockdown increased LC3B-II levels in both PC3 and SKOV3 cells. **c** TEM images of PC3 cells treated with AEE788 compared to cells treated with EGFR siRNA (multimembranous non-selective autophagosomes are indicated by arrows and high electronic density mitochondria fragment containing mitophagosomes are indicated by arrow heads). **d** TEM images of SKOV3 cells treated with AEE788 compared to cells treated with EGFR siRNA (multimembranous non-selective autophagosomes are indicated by arrows and high electronic density mitochondria fragment containing mitophagosomes are indicated by arrow heads). **e** Quantification of non-selective autophagosomes (open bar) and mitophagosomes (gray bar) of data in **c** (>20 cells from seven randomly selected areas of each sample were counted, * indicates statistical significance compared to control cells, *n* ≥ 3). **f** Quantification of non-selective autophagosomes (open bar) and mitophagosomes (gray bar) of data in **c** (>20 cells from seven randomly selected areas of each sample were counted, * indicates statistical significance compared to control cells, *n* ≥ 3)

We investigated the processes of TKI (AEE788)-induced autophagy, and autophagy induced by siRNA-mediated knockdown of EGFR protein on two types of cancer cells (prostate cancer PC3 cells and ovarian cancer SKOV3 cells). We observed that the autophagic responses to these two treatments were, in fact, completely different. Both AEE788 treatment and EGFR knockdown showed similar upregulation the autophagic protein, LC3B-II^13^ (Fig. 1a, b); however, transmission electronic microscopy (TEM) imaging revealed that AEE788 caused non-selective autophagy characterized by the accumulation of autophagosomes devoid of mitochondria, whereas EGFR knockdown led to selective mitophagy, characterized by the presence of mitophagosomes containing electron-dense mitochondrial fragments and a corresponding depletion of cytosolic mitochondria (Fig. 1c, f). These data suggest that the EGFR protein, but not its tyrosine kinase activity, is required to suppress mitophagy.

### The mTORC2/Akt axis is differentially regulated by EGFR’s kinase-dependent and -independent functions

**Fig. 2:**
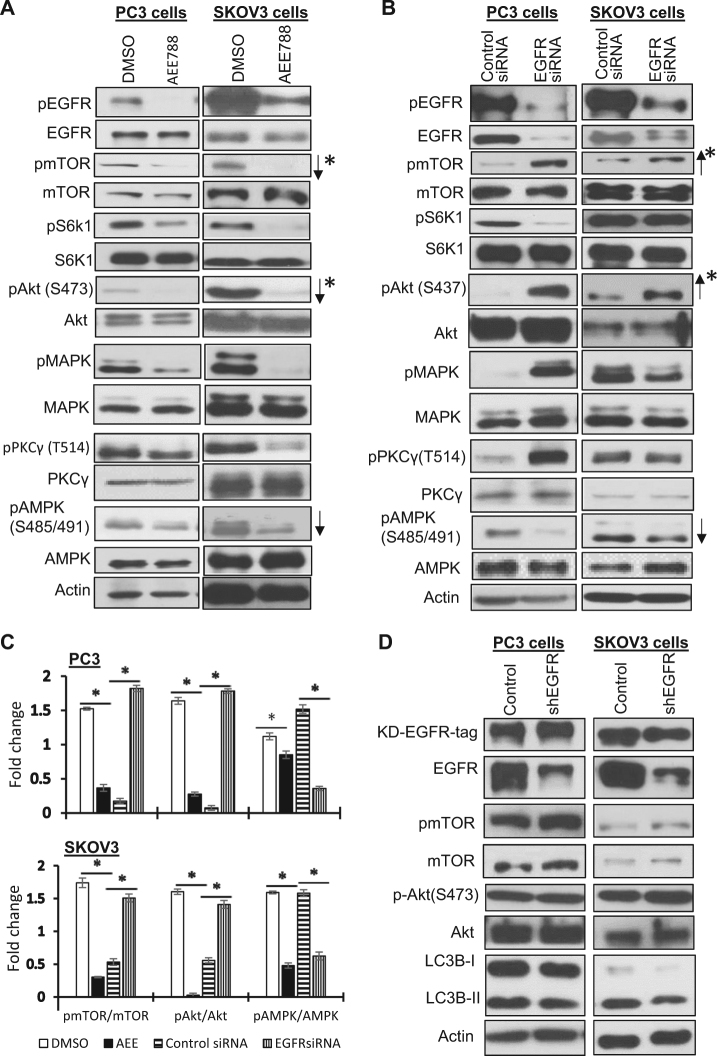
Comparison of signaling pathways affected by inhibition of EGFR’s kinase and EGFR knockdown. **a** Western blot analysis of signaling pathway proteins upon inhibition of EGFR by AEE788 (5 μM) for 24 h (arrows indicating pathways similarly altered in both PC3 and SKOV3 cells). **b** Western blot analysis of signaling pathway proteins upon EGFR knockdown by siRNA. (Arrows indicating pathways similarly altered in both PC3 and SKOV3 cells). **c** Quantification of Western blot results of mTOR, Akt, and AMPK pathway shown in **a** and **b** (* denotes *P* < 0.01; Student’s *t*-test). Each target band was normalized to its internal control. All immunoblots are representative of at least three experimental repeats. **d** Western blot analysis of mTOR, Akt, LC3B-I and II of cells overexpressing with an HA-tagged kinase-dead EGFR (R817M mutant) in response to knockdown of endogenous EGFR by shRNA targeting the 5′-UTR of endogenous EGFR mRNA. Note: pmTOR and pAkt levels are oppositely changed by AEE788 and EGFR siRNA in both types of cells, however AMPK activity was similarly altered by AEE788 and EGFR siRNA

To determine the signaling pathways mediating the loss-of-EGFR-induced mitophagy, we compared the effects of AEE788 and EGFR siRNA treatment of PC3 and SKOV3 cells on the activity of several protein kinases related to cell survival, including Akt, MAPK, AMPK, PKCγ, and mTOR (mTORC1 and mTORC2 pathways) (Fig. 2a, c). In both cell lines, AEE788 and EGFR knockdown showed similar effects on AMPK and mTORC1, activation of AMPK as evidenced by reduction in S485/S491 phosphorylation^14^, and inhibition of mTORC1 activity as indicated by decrease of S6K1 phosphorylation^15^. However, these treatments exerted opposite effects on mTOR and Akt, these two kinases were inhibited by AEE788, whereas loss-of-EGFR activated them. In PC3 cells, AEE788 treatment and EGFR knockdown also exhibited opposite effects on pPKCγ (T514) and pMAPK (inhibited by AEE788 and activated by EGFR siRNA) and similar effects on pS6K1 (reduced by both treatments). However, these effects were not observed in SKOV3 cells, suggesting PKCγ, MAPK, and mTROC1 are not critically involved in loss-of-EGFR-induced mitophagy, and further, that the responses of these signaling pathways to EGFR alterations are cell type dependent. To further determine the kinase-independent role of EGFR in regulating mTORC2 and Akt activity, we knocked down EGFR in cells overexpressing an HA-tagged kinase-dead EGFR (the R817M mutant)^16^. As shown in Fig. 2d, knockdown of endogenous EGFR by shRNA targeting the 5′-UTR of endogenous EGFR failed to activate mTOR or Akt, and most importantly failed to upregulate LC3B-II, supporting that EGFR can suppress the mTORC2/Akt pathway and autophagy independent of EGFR’s kinase activity.

### EGFR interacts and stabilizes the upstream of mTORC2 (UT2) independent of EGFR’s kinase activity

**Fig. 3:**
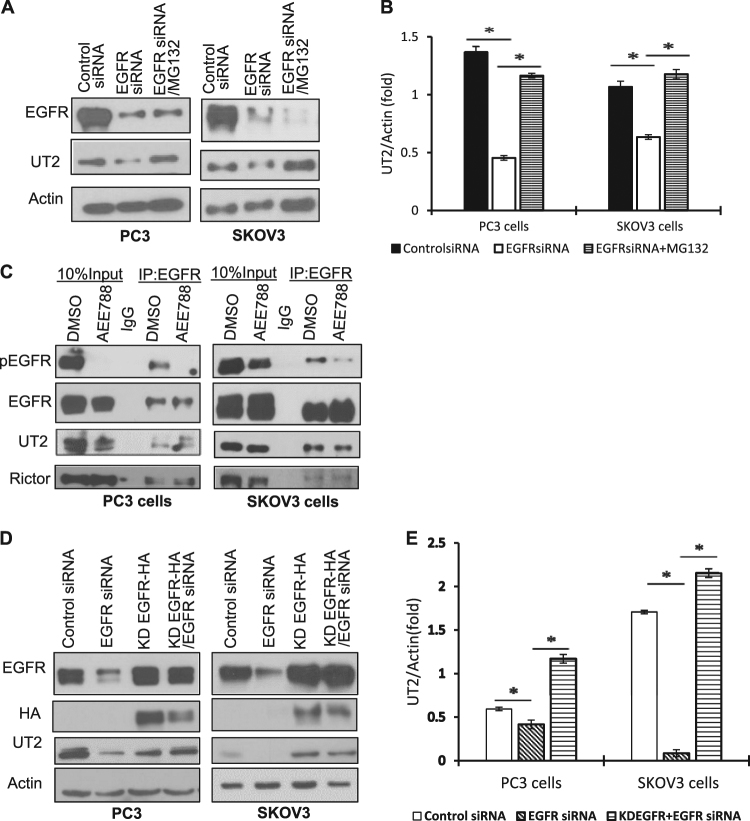
EGFR interacts and stabilizes the upstream of mTORC2 (UT2) independent of EGFR’s kinase activity. **a** Knockdown of EGFR decreased UT2, which could be inhibited by a proteasome inhibitor MG132. **b** Quantification of Western blot results of UT2 shown in **a** (* denotes *P* < 0.01; Student’s *t*-test). Each target band was normalized to beta actin control. All immunoblots are representative of at least three experimental repeats. **c** UT2 and RICTOR was co-immunoprecipated with EGFR regardless of AEE788 (10% input, 10% of the amount of proteins used for the immunoprecipitation experiments). **d** Kinase-dead EGFR (KD-EGFR) inhibited EGFR knockdown-induced downregulation of UT2. **e** Quantification of Western blot results of UT2 shown in **d** (* denotes *P* < 0.01; Student’s *t*-test). Each target band was normalized to beta actin control. All immunoblots are representative of at least three experimental repeats

As it is known that Akt is the major intracellular target of mTORC2^15^, and mTOR is activated by loss-of-EGFR, we hypothesized that activation of mTORC2 signaling might be responsible for loss-of-EGFR-induced mitophagy. In determining the mechanism underlying mTORC2 activation by loss-of-EGFR, we turned our attention to the upstream of mTORC2 (UT2), an integral membrane protein that represses mTORC2 activity^17^. To determine the role of UT2 in loss-of-EGFR-induced mTORC2 activation, we performed a series of experiments. As shown in Fig. 3a, b, EGFR knockdown significantly decreased the level of UT2, and this effect was inhibited by a proteasome inhibitor, MG132, suggesting that loss-of-EGFR destabilizes UT2. To further determine whether EGFR physically interacts with UT2 independent of EGFR’s kinase activity, we immunoprecipitated EGFR from both intact and AEE788-treated cells, and probed the precipitates for UT2 and RICTOR (a specific component of the mTORC2) using Western blot analysis. We found that both UT2 and RICTOR were co-precipitated with EGFR in both untreated and AEE788-treated cells (Fig. 3c), suggesting that EGFR interacts with UT2 and RICTOR independent of EGFR’s tyrosine kinase activity and can repress mTORC2 by interacting and stabilizing UT2. To further test this possibility, we treated cells with EGFR shRNA targeting the 5′-UTR of endogenous EGFR^7^, followed by overexpression of the HA-tagged kinase-dead EGFR (R817M). As shown in Fig. 3d, e, expression of the kinase-dead EGFR inhibited the downregulation of UT2 caused by EGFR knockdown, supporting that EGFR stabilizes UT2 and inhibits mTORC2 independent of EGFR’s kinase activity. Together, these data suggest that EGFR represses mTORC2 activity by interacting with and stabilizing UT2 independent of EGFR’s kinase activity.

### Inhibition of Akt or mTORC2 blocked loss-of-EGFR-induced mitophagy

**Fig. 4:**
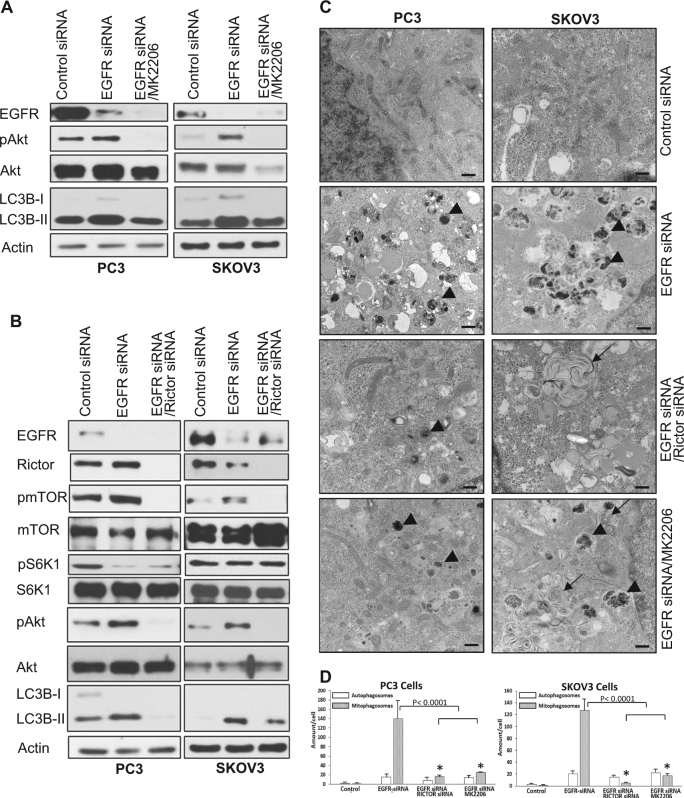
Inhibition of Akt or mTORC2 blocked loss-of-EGFR-induced mitophagy. **a** An Akt inhibitor, MK2206, was efficient of inhibiting EGFR knockdown-induced Akt activation and LC3B-II elevation in both PC3 and SKOV3 cells. **b** Effects of Rictor knockdown on loss-of-EGFR-induced activation of mTOR, Akt, and upregulation of LC3B-II. **c** TEM images of PC3 and SKOV3 cells treated with control siRNA, EGFR siRNA ± MK2206, or ± Rictor siRNA. **d** Quantification of non-selective autophagosomes and mitophagosomes of data shown in **c** (* indicates statistical significance compared to control cells, *n* ≥ 3)

It is known that mTORC2 activates Akt^18^. To further elucidate the role of mTORC2 and Akt in the loss-of-EGFR-induced mitophagy, we treated EGFR knockdown PC3 and SKOV3 cells with either a specific Akt inhibitor, MK2206, or with Rictor siRNA, to inhibit Akt and mTROC2, respectively. As shown in Fig. 4a, MK2206 blocked EGFR knockdown-induced Akt activation and induction of LC3B-II, and knockdown Rictor inhibited loss-of-EGFR-induced activation of mTOR and Akt as well as induction of LC3B-II. These results suggested that mTORC2 and Akt activation are required for loss-of-EGFR-induced autophagy. TEM imaging revealed that inhibition of Akt or mTORC2 was sufficient in inhibiting loss-of-EGFR-induced selective mitophagy (Fig. 4c, d).

### Activation of mTORC2-induced mitophagy

**Fig. 5:**
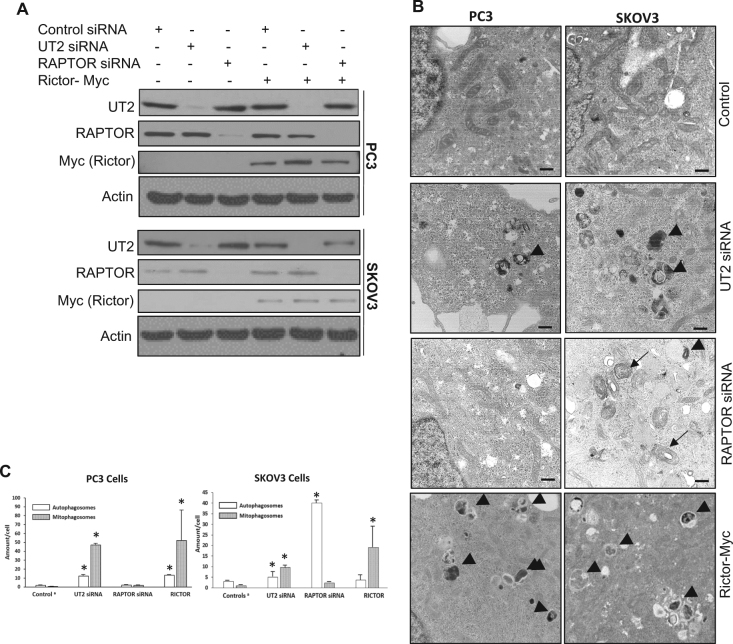
Activation of mTORC2 is sufficient to induce selective mitophagy. **a** Western blot analysis confirming sufficient knockdown of UT2, RAPTOR, and overexpression of Rictor-myc in both PC3 and SKOV3 cells. **b** TEM images of PC3 and SKOV3 cells treated with control vector (for the RICTOR-myc group), UT2 siRNA, RAPTOR siRNA, and RICTOR-myc expressing plasmid (mitophagosomes are indicated by arrow heads, non-selective autophagosomes are indicated by arrows). **c** Quantification of non-selective autophagosomes and mitophagosomes of data shown in **b** (* indicates statistical significance compared to control cells, *n* ≥ 3), (**a** because the vector transfected control cells and control siRNA treated cells are similar, data of these control groups were pooled as control values for all the other experimental groups)

To further determine the role of mTORC2 in regulating mitophagy, we activated mTORC2 either by knockdown of UT2 or overexpression of Rictor in EGFR intact cells. In parallel, we also knocked down a key mTORC1 component, Raptor, as a control. The sufficiency of inhibition of Akt and mTORC2, knockdown of UT2, Rictor overexpression, and Raptor knockdown is shown in Fig. 5a–c shows that activation of mTROC2 was sufficient to induce selective mitophagy, although these cells exhibited differences in response to specific mTORC1 inhibition caused by Raptor knockdown (it caused significant level of non-selective autophagy in SKOV3 cells but not in PC3 cells).

### An EGFR-downregulating peptide, Herdegradin, induced alterations in signal pathways similar to EGFR knockdown, killed cancer cells and induced mitophagy in vitro, and induced mitophagy and inhibited growth of orthotopic SKOV3 cancers in vivo

**Fig. 6:**
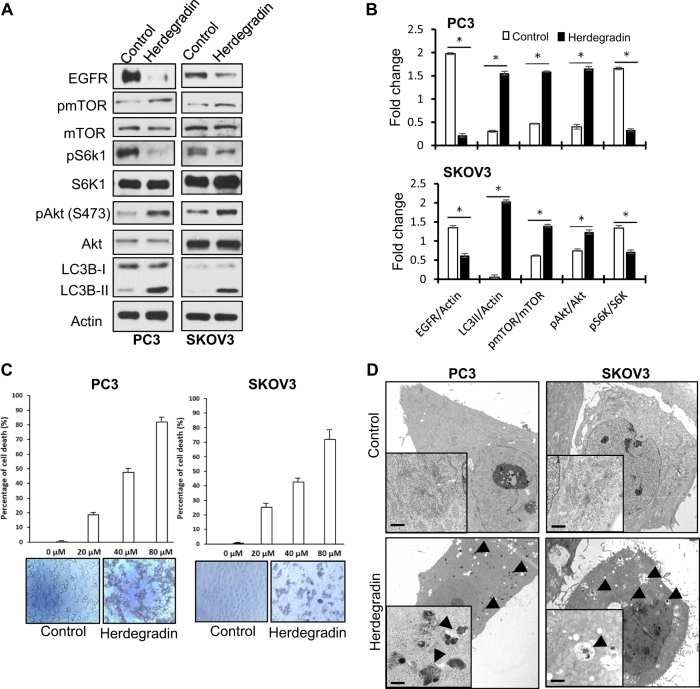
An EGFR-downregulating peptide, Herdegradin, induced alterations in signal pathways similar to EGFR knockdown, killed cancer cells, and induced mitophagy in vitro. **a** PC3 and SKOV3 cells were treated with Hedegradin (50 μM) for 24 h. Western blot analysis revealed that EGFR was decreased, mTOR was activated, phosphorylation of S6K1 was decreased, and Akt was activated, and LC3B-II was upregulated. **b** Quantification of Western blot results of EGFR, LC3B-II, pmTOR, pAkt, and pS6K1 shown in **a** (* denotes *P* < 0.01; Student’s *t*-test). Each target band was normalized to either beta actin control (EGFR and LC3B-II) or to its internal total protein control (mTOR, Akt, and S6K1). All immunoblots are representative of at least three experimental repeats. **c** Herdegradin induced cell death in both PC3 and SKOV3 cells in a dose-dependent manner analyzed by Trypan blue uptake assay (treatments were done at indicated dosages for 48 h, *n* = 6 in each group). **d** TEM images showed that Herdegradin induced mitophagy in both PC3 and SKOV3 cells after 72 h of treatment

**Fig. 7:**
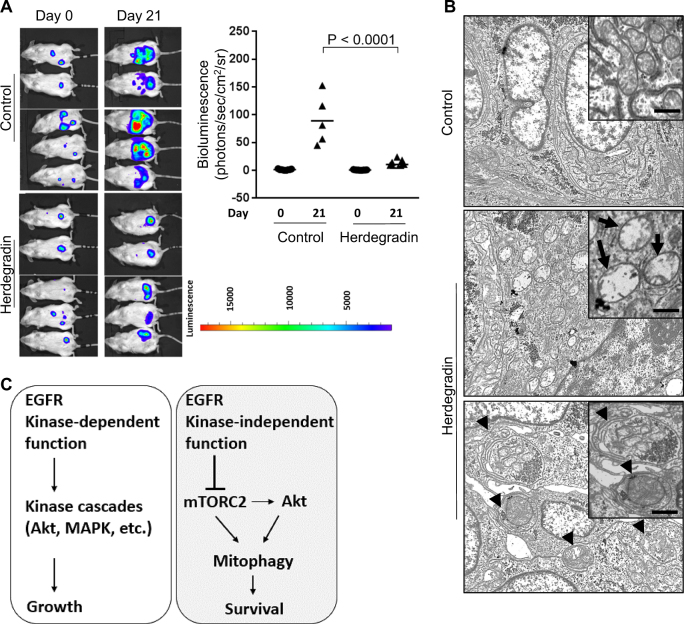
An EGFR-downregulating peptide, Herdegradin, inhibited growth and induced mitophagy of orthotopic SKOV3 cancers in vivo. **a** Herdegradin the growth of orthotopic SKOV3 cancers in vivo, which was evidenced by in vivo imaging. **b** TEM images revealed that Herdegradin caused significant damage to the mitochondria (loss of cristae, arrows) and induced mitophagy (arrow heads). **c** Schematic summary of the kinase-independent anti-mitophagy function of EGFR in comparison to its canonical tyrosine kinase-dependent functions. In this model, EGFR represses mitophagy by inhibiting the mTORC2/Akt axis independent of EGFR’s tyrosine kinase activity, and unlike the kinase-dependent functions of EGFR that regulate cell growth by activating its downstream kinase cascades, the kinase-independent function of EGFR predominantly controls the survival of cancer cells by repressing mitophagy

Previously, we have shown that EGFR and the sodium/glucose co-transporter 1 mutually stabilize one another by interacting at EGFR’s carboxyl tail^7,19^, which offers a primary targetable region to destabilize EGFR. We designed and tested a series of short peptides mimicking segments of the carboxyl tail region of EGFR. We found that a 14-amino-acid peptide composed of D-amino acids corresponding to the amino acids 1049–1062 of a human EGFR (GenBank: AAH94761.1) was able to downregulate EGFR protein, activate mTOR and Akt, and upregulate LC3B-II (Fig. 6a, b). We named this EGFR-downregulating peptide Herdegradin. Herdegradin exhibited cytotoxicity to both PC3 and SKOV3 cancer cells in vitro (Fig. 6c). Transmission electronic microscopy (TEM) imaging revealed that Herdegradin caused massive mitophagy in both PC3 and SKOV3 cells within 24 h of treatment (Fig. 6d). Similar results were observed in two other cancer cell lines, A549 (lung cancer) and HCT116 (colon cancer) (Fig. S1). To assess a potential therapeutic effect of Herdegradin in vivo, we employed the orthotopic ovarian cancer model, inoculating luciferase expressing SKOV3 cells into the abdominal cavity of female SCID mice^20^. Two weeks after inoculation of cancer cells, we administrated Herdegradin at a dose of 3 mg/kg/day (i.p.) for 21 days. Tumor progression was monitored weekly by in vivo imaging of luciferase. As shown in Fig. 7a, in vivo live imaging found that Herdegradin significantly inhibited tumor progression, and the tumor load was also validated at time of biopsy at the end of in vivo experiments (Fig. S3). Furthermore, TEM revealed that Herdegradin caused severe damage to the mitochondria and induced mitophagy, which were not observed in the control samples (Fig. 7b). Basing on the data presented by this study, we propose a novel mechanism by which EGFR represses mitophagy via inhibiting the mTROC2/Akt axis independently of EGFR’s tyrosine kinase activity (Fig. 7c).

## Discussion

EGFR is a major therapeutic target for cancer treatment. Two classes of therapeutic agents have been developed, monoclonal antibodies that block the binding of EGFR activating ligands, and small molecules of TKIs that reversibly or irreversibly occupy the ATP binding pocket of EGFR. However, the therapeutic efficacy of these EGFR inhibitors has been disappointing. Most cancers of epithelial origin express or overexpress EGFR^21,22^. However, only a few types of cancer, such as non-small cell lung cancer (NSCLC) and KRAS wild-type colorectal cancer, exhibit significant, but transient, effectiveness^1^. Furthermore, for even the most responsive cancers, the NSCLC, only those with gain-of-function mutations in EGFR tyrosine kinase activity respond well to TKIs, whereas cancers that express wild-type EGFR either respond poorly, or not at all^23^. Finally, without exception, cancers that initially respond to TKIs develop acquired resistance to the drugs, often within a few months of treatment^24^. We have previously reported that EGFR possesses kinase-independent pro-survival functions^7,19,25–27^, a hypothesis that is also supported by recent reports from other laboratories^8,9^. The KID functions of EGFR offer a new window for targeting EGFR expressing cancers.

The current study identifies a novel mechanism and signal pathway underlying EGFR’s KID function(s), inhibition of mitophagy via repression of the mTORC2/Akt pathway (summarized in Fig. 7c). The differential responses of PKC, mTORC1, and MAPK of the two types of cancer cells to loss-of-EGFR indicate that the KID functions of regulating these pathways are cell type dependent, and the commonality of mTORC2/Akt in mediating loss-of-EGFR-induced mitophagy among the different cell types argues that this EGFR’s kinase-independent anti-mitophagy pathway is a more fundamental mechanism. Our data are consistent with the observation that Akt activation is also pro-mitophagic in macrophage^28^. It is intriguing that Akt can be activated by membranous growth factor receptors such as EGFR^29^ and intracellularly by mTORC2^18^, however these different routes of Akt activation lead to complete different cellular responses, cell growth/survival by the former route and mitophagy/cell death by the later one. The activation of mTORC2 upon loss-of-EGFR protein explains the Akt activation in response to EGFR knockdown that we observed previously^7^. The mechanism underlying the differential roles of Akt merits further investigation.

In this study, for the first time, we have shown that EGFR suppresses mitophagy by a mechanism that is independent of its tyrosine kinase activity, and activation of mTORC2 induces mitophagy in cancer cells. In addition, we report Herdegradin, a synthetic peptide that is capable of downregulating EGFR, activating mTORC2, and inducing mitophagic cell death in a manner that is similar to the mitophagic cell death caused by EGFR knockdown. The mTOR kinase is a component of two distinct protein kinase complexes, mTORC1 and mTORC2. Although much is known about the function of mTORC1, our knowledge of the biological role of mTORC2 is limited^15^. It is known that mTORC1 stimulates mitochondrial activity and biogenesis^30,31^, and we show in the current study that activation of mTORC2 promotes mitophagy. The mTORC1 pathway is anti-autophagic and anti-mitophagic via inhibiting some downstream autophagy related proteins, such as the Unc-51-like kinase 1 (ULK1), which are otherwise activated by pro-autophagic mechanisms^32–34^. Studies have shown that phosphorylation of the ULK1 protein at serine 555 by AMPK is pro-autophagic and pro-mitophagic in response to nutrient starvation^35^ or hypoxia^36^, whereas phosphorylation at the serine 757 site by mTOR1 is anti-autophagic and anti-mitophagic^35^. In the current study, we found that loss-of-EGFR either induced by siRNA or by Herdegradin decreased the levels of S555-phosphorylated ULK1, whereas increased the levels of S757-phosphorylated ULK1 (Fig. S2), which is opposite to the phosphorylation changes of ULK1 responding to starvation or hypoxia-induced autophagy, but it is consistent with the recent finding that the S757 of ULK1 can also be phosphorylated by AKT^37^ given that AKT is activated by loss-of-EGFR, and suggests that ULK1 might be uniquely involved in the loss-of-EGFR-induced mitophagy, however its role needs to be defined by further studies.

Although several studies have shown that mTORC1 often represses mTORC2 activity^38^, developing dual-inhibitors for both mTORC1 and mTORC2 has been a major approach of targeting the mTOR pathways for cancer treatment, and these inhibitors have exhibited limited clinical benefits^39^. Given the opposing roles of mTORC1 and mTORC2 on the fate of mitochondria, it is proposed that concurrent inhibition of mTORC1 and activation of mTORC2 might be a better strategy for cancer treatment, however specific mTORC2 activators are yet to be developed. Our EGFR-downregulating peptide showed potent effects on mTORC1 inhibition and mTORC2 activation, it may serve as a valuable tool for developing specific mTORC2 activators.

Our previous studies have shown that EGFR can exist in two statuses in cancer cells, a kinase responsive status that governs the classical EGFR’s kinase-dependent functions and a kinase-independent status that maintains cell survival by interacting with crucial pro-survival proteins such the sodium/glucose co-transporter 1^19^. The repression of mTORC2 by KID function of EGFR shown herein adds another functional component to the kinase-independent status of EGFR. The KID functions of EGFR might be a critical survival node for cancers that overexpress wild-type EGFR as these cancers are innately resistant to EGFR TKIs, where the KID functions of EGFR are elevated due to overexpression, and for cancers that have acquired resistance to EGFR TKIs, where EGFR’s function has been shifted to its KID functions by TKIs. Co-targeting EGFR’s KD and KID functions may hold a new promise of treating EGFR-positive cancers.

## Materials and methods

### Cell culture

Human Prostate cancer cell line PC3 and Ovarian cancer cell line SKOV3, were obtained from ATCC. These cells were cultured in DMEM medium containing 5 mM glucose and supplemented with 10% fetal bovine serum along with antibiotics in a CO_2_ cell culture incubator.

### Antibodies and common reagents

The following antibodies were used: EGFR (sc-03-G), Akt1 (sc-1618), C14ORF37 (UT2) (sc-139226), Rictor (sc-271081), Raptor (sc-81537) pAMPK (S485/491), AMPK (sc-25793), Alpha-Tubulin (sc-5546), were purchased from Santa Cruz Biotechnology. pEGFR (Y1173) from Invitrogen, Myc-tag (Cat#2278S), mTOR (Cat#2972), phospho p70S6K1 (T389) (Cat#9204), pPKC (T514) (Cat#9379), pAkt (S243) (Cat#4060), phospho MAPK (Cat#9101S), MAPK (Cat#4695), ULK1 (Cat#8054), pULK1-S757 (Cat#6888), and pULK1-(Cat#5869) were purchased from Cell Signaling. Actin (Cat#A2228) was from Sigma. PKC (Cat# ab71558) and phospho-mTOR(Cat#ab109268) were from Abcam. C225 (Cat #MABF120), was from EMD Millipore for EGFR immunoprecipitation studies. LC3 (Cat#NB100-2220) was from Novus biologicals. EGFR TKI AEE788 (Cat# S1486), Akt inhibitor, MK2206 (Cat#S1078), were obtained from Selleckchem. Plasmid-based transfections and siRNA-based transfections were performed using Lipofectamine 3000 (Invitrogen) and Lipofectamine RNAiMax (Invitrogen), respectively. Protein A/G beads (Santa Cruz Biotechnology) were used for immunoprecipitation.

### Peptide

The EGFR-downregulating peptide, Herdegradin, composed of d-amino acids, LVWKQSCSSTSSTH, was synthesized by Genscript Inc. at purity >98.0%. Cell culture medium containing 10% FBS was used to prepare fresh peptide solution for each set of experiment.

### Plasmids and siRNAs

pRNAT-U6.1/Neo vector from GenScript was used to generate vector-based shRNA against EGFR. The target sequence of shEGFR targeting the 5′-UTR of human EGFR was CTGACTCCGTCCAGTATTGAT and negative control shRNA sequence was GAACAATGTTGACCAGGTGA. Plasmid containing kinase-dead EGFR (KD-EGFR (R817M) was inserted into pcDNA3.1 vector as described in our previous paper. siRNAs for EGFR (Cat#EHU076761), RAPTOR (SiRNA ID: SASI_Hs01_00048380) and UT2 (c14orf37) (SiRNA ID: SASI_Hs01_00126164) were purchased from Sigma Aldrich. Rictor siRNA (sc-61478) was purchased from Santa Cruz Biotechnology. Rictor-myc expressing and control vectors were from Dr. Dos Sarbassov of the MD Anderson Cancer Center.

### Transfection, immunoprecipitation, and western blot

PC3 and SKOV3 cells were transfected using Lipofectamine 3000 and Lipofectamine RNAiMax according to the protocol recommended by manufacturer (Invitrogen).To knockdown EGFR from PC3 and SKOV3 cells, 2 μg of shRNA for EGFR plasmid or 400 ng of EGFR siRNA (from Sigma Aldrich) was used per well for 12-well plate to transfect using Lipofectamine 3000 or Lipofectamine RNAiMax. Protein samples were collected for PC3 and SKOV3 cells after 72 and 48 h, respectively.

PC3 and SKOV3 cells were lysed using CHAPS-Lysis buffer (40 mM HEPES, pH 7.4, 120 mM NaCl, 2 mM EDTA, 0.3% CHAPS) and incubated with anti-EGFR antibody and protein A/G beads overnight at 4 °C on shaker. Antibody bound beads were pelleted by centrifugation at 5000 rpm for 2 min at 4 °C. Subsequently, the beads were washed five times using CHAPS-lysis buffer by centrifugation. 2×sample Lammelli buffer was added to the precipitated beads and boiled at 100 °C for 5 min.

Equal amount of protein samples were run on SDS-PAGE and transferred to PVDF membrane followed by blocking in 5% milk in TBST buffer for 1 h. Then it was incubated with primary antibodies in blocking buffer overnight at 4 °C. Later it was washed in TBST buffer for three times 10 min each. Afterwards, HRP-conjugated secondary antibody was added at 1:3000 dilution for 1 h at room temperature. Followed by washing in TBST buffer for three times, the membranes were exposed to ECL solution. Protein signals were detected on the auto radiographic films and signal intensities of at least three experimental repeats were quantified by using the software Image J.

### Trypan blue assay

Live cells were stained by trypan blue (0.2% in PBS) for 3 min in a cell culture incubator before fixed by 0.4% PFA for 3 min at room temperature. Cells were then briefly washed with PBS and visualized under an inverted microscope. Live and dead cells (blue cells) were counted in three randomly selected fields containing more than 200 cells for each sample (*n* = 3 in each group).

### In vivo tumor development assay

Female *NOD.Cg-Prkdc*^*scid*^B2m^tm1^/*Nju* mice of 4 weeks of age were obtained from Nanjing Biomedical Research Institute of Nanjing University. The mice were maintained under specific pathogen-free conditions in facilities approved by the American Association for Accreditation of Laboratory Animal Care and in accordance with current regulations and standards institutional guidelines. To track the location and proliferation of skov3 cells in vivo, cells were labeled with FFLuc (skov3-FFluc). For the production of tumors, the cells growing in culture were harvested by a brief treatment with 0.25% trypsin and 0.02% EDTA. A single-cell suspension of 5 × 10^5^ cells with a viability of >95% was injected into the peritoneal cavity of the mice. The mice were monitored daily for evidence of disease (abdominal swelling, hunched posture, and listlessness) and live imaging of development of tumor was performed weekly using the in vivo imaging system (IVIS Lumina II). Two weeks after tumor cell inoculation, mice were divided into two groups bearing similar tumor load before treatment with either equal volume of vehicle (saline) or Herdegradin (3 mg/kg/day) for consecutive 21 days, and tumor development was imaged weekly. At the end of treatment, mice were killed for tissue collection, and tumor tissues were fixed for TEM analysis.

### Transmission electron microscope imaging

For TEM imaging, cells were washed in PBS buffer for three times and were fixed in fixative buffer (3% glutaraldehyde, 2% paraformaldehyde in 0.1 M cacodylate buffer (pH 7.3)). Images were taken by TEM. TEM imaging was performed in the High Resolution Electronic Microscopy Facility at M.D. Anderson Cancer Center. For quantification of autophagoisomes and mitophagosomes, there were more than 60 cells from seven randomly selected areas of each sample counted, and there are three samples in each experimental group, which gives 180 cells used for each group.

### Statistical analysis

Investigators who analyzed the data are blinded from experimental grouping. The Student’s two-sided *t*-test was used to assess the difference of values of control and individual experimental groups. Variances between compared groups are similar. Data are presented as means ± S.D., and *P* < 0.05 is defined as statistical significance.

## Electronic supplementary material

Figure S3

Figure s2

Figure S1
