# Supplementary material for: Targeted reduction of the EGFR protein, but not inhibition of its kinase activity, induces mitophagy and death of cancer cells through activation of mTORC2 and Akt
Source: Oncogenesis. 2018 Jan 23;7(1):5. doi: 10.1038/s41389-017-0021-7 (PMC5833766; doi:10.1038/s41389-017-0021-7)
Supplement: Supplementary file 1 — Figure S3 [file 41389_2017_21_MOESM1_ESM.pdf]

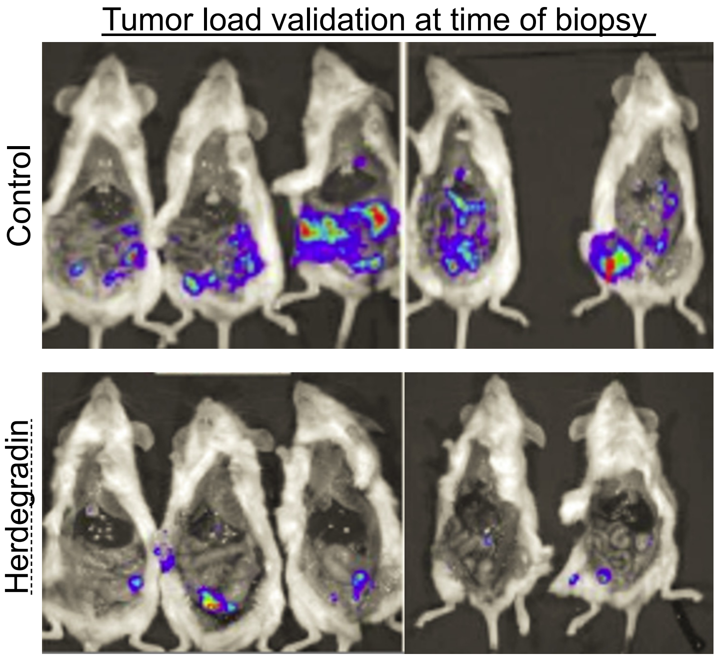

Figure S3. Validation of tumor load.

The tumor load measured by in vivo live-imaging shown in Figure 7A was validated at time of biopsy at the end of in vivo experiment. The result is consistent with the live-imaging data.
