# Supplementary material for: Targeted reduction of the EGFR protein, but not inhibition of its kinase activity, induces mitophagy and death of cancer cells through activation of mTORC2 and Akt
Source: Oncogenesis. 2018 Jan 23;7(1):5. doi: 10.1038/s41389-017-0021-7 (PMC5833766; doi:10.1038/s41389-017-0021-7)
Supplement: Supplementary file 2 — Figure s2 [file 41389_2017_21_MOESM2_ESM.pdf]

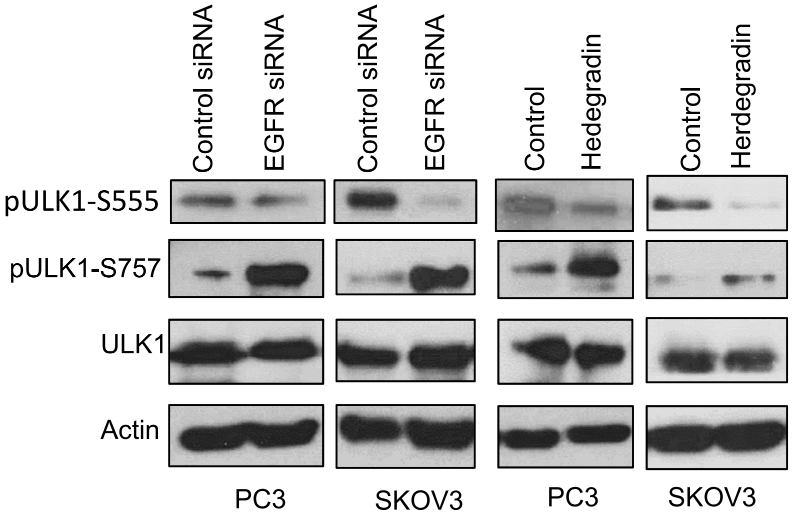

Figure S2. Loss-of-EGFR, induced by siRNA or by the EGFR-downregulating peptide, Herdegradin, decreased the level of serine 555 phosphorylated ULK1 (pULK1-S555) and increased the serine 757 phosphorylated ULK1 (pULK1-S757) in both PC3 and SKOV3 cells.
