# Supplementary material for: Targeted reduction of the EGFR protein, but not inhibition of its kinase activity, induces mitophagy and death of cancer cells through activation of mTORC2 and Akt
Source: Oncogenesis. 2018 Jan 23;7(1):5. doi: 10.1038/s41389-017-0021-7 (PMC5833766; doi:10.1038/s41389-017-0021-7)
Supplement: Supplementary file 3 — Figure S1 [file 41389_2017_21_MOESM3_ESM.pdf]

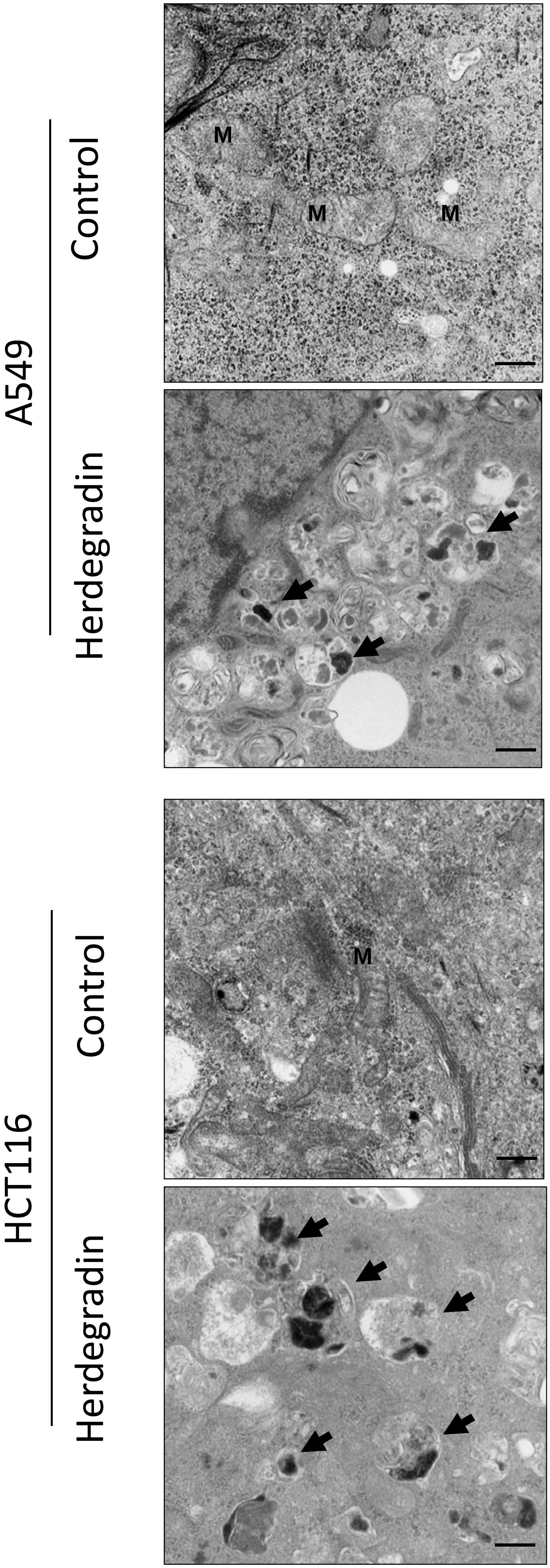

Figure S1.

The EGFR-downregulating peptide, Herdegradin, caused massive mitophagy in A549 and HCT116 cells.

Cell were treated with Herdegradin for 6hr at a dosage of 100 µM in the presence of 10% FBS in the medium before being processed for TEM imaging. (Mitophagosomes are Indicated by arrowheads. Healthy mitochondria are marked with letter M. Bar=500nm).

Figure S1
